# 

**Published:** 1896-07-11

**Authors:** 


					THE HOSPITAL; ABSOLUTELY PURE, therefore BEST,
July 11th, 1896.
OADo?H?Y's Pt H *? CADJ?F'*?
" ^tainM^^?lfaMet.St pUrit^ at presenl MM JIS^? NO ALKALIES USED. fettAox
nI^^xx? with extra nursing supplement. HSffiSP
Saturday, July 11th, 1896. [R^S?rio^te^toe^S'tKS5dom SllKSS "*

				

